# StudentNet: an edge-deployable approach for student behavior detection in smart classrooms

**DOI:** 10.3389/fncom.2026.1828972

**Published:** 2026-07-09

**Authors:** Lei Ai, Huihui Hu

**Affiliations:** Marxism School, Pingdingshan University, Henan, China

**Keywords:** behavior recognition, deep learning, edge deployment, object detection, smart education, YOLO

## Abstract

**Introduction:**

Accurately identifying student behavior in classroom environments is essential for the development of intelligent education systems. However, existing methods often exhibit high false-positive and false-negative rates in complex backgrounds or densely populated scenes, which degrades detection accuracy.

**Methods:**

To overcome these limitations, we propose StudentNet, a novel framework incorporating the Behavior-Enhanced Fusion Super-Resolution Network (BEF-SRNet), the Direction-Sensitive Adaptive Convolution module (DSAC), and the Adaptive Spatial Feature Fusion (ASFF) strategy. BEF-SRNet separates regions of interest from background clutter to improve the discriminability of behavioral features. DSAC uses multi-directional branches and channel attention to extract structural behavioral cues while suppressing noise. ASFF enhances scale-invariant feature representation to improve semantic consistency and detection performance.

**Results:**

Experiments on SCB-Dataset3 show that StudentNet improves mAP@50 and F1-score by 6.3 and 6.4 percentage points, respectively, compared with the YOLOv8n baseline. Deployment on the Core-3399Pro-JD4 edge platform further shows that the INT8-quantized model achieves 38.4 FPS with acceptable accuracy degradation.

**Discussion:**

These results indicate that StudentNet improves student behavior detection in complex classroom scenes and is suitable for real-time smart classroom applications on edge devices.

## 1 Introduction

The proliferation of artificial intelligence has catalyzed a paradigm shift in modern education, driving a transition from traditional pedagogy to data-driven intelligent instruction (Dimitriadou and Lanitis, 2023). Within this ecosystem, the automated analysis of student behavior serves as a cornerstone for evaluating classroom engagement, identifying academic anomalies, and facilitating personalized teaching strategies (Yang et al., 2020; Li et al., 2023). By leveraging computer vision techniques to analyze non-intrusive video streams, educators can obtain fine-grained insights into learning states such as reading, writing, and hand-raising, thereby enabling timely interventions and evidence-based classroom management (Ngoc Anh et al., 2019; Trabelsi et al., 2023).

In recent years, deep learning-based object detection frameworks have achieved remarkable success in general scenarios (Zou et al., 2023; Ragab et al., 2024). YOLO-series detectors, in particular, have been widely adopted in educational applications because they offer a favorable balance between speed and accuracy (Yin et al., 2022; Zhou et al., 2022). However, although these general-purpose models perform well in detecting distinct objects in open environments, their direct application to complex classroom scenes remains suboptimal (Buono et al., 2023).

The challenge of student behavior detection is multifaceted and distinct from general object detection tasks. First, classrooms are characterized by severe occlusion and visual clutter. This complexity causes standard convolutions to fail to disentangle foreground targets. Second, there is an extreme scale imbalance. Students in the front row occupy large pixel areas, while those in the back rows appear as small, low-resolution targets, often as small as 45 × 45 pixels, leading to high miss rates for distant subjects. Third, student behaviors exhibit complex, non-rigid pose variations. Distinguishing between similar actions, such as “writing” and “reading,” involves subtle structural differences that are direction-dependent. Standard isotropic convolutions frequently struggle to capture these nuances, resulting in semantic ambiguity and poor localization in densely populated scenes.

To address these challenges, numerous studies have explored deep learning-based approaches for student behavior detection in classroom settings. Chen et al. (2023) enhanced YOLOv8 by incorporating the multi-scale attention module EMA and a multi-head self-attention mechanism to improve detection accuracy in occluded scenarios, achieving a 4.2% improvement in mAP@50 on the SCB-Dataset. Zhu and Yang (2024) proposed CSB-YOLO, a lightweight real-time algorithm with only 0.72M parameters, demonstrating strong performance on resource-constrained classroom devices. Jia and He (2024) developed a student behavior detection model combining YOLOv5 with contextual attention and OpenPose, achieving 82.1% mAP in complex classroom environments. Han et al. (2025) introduced WAD-YOLOv8, incorporating weighted attention mechanisms to enhance multi-scale feature extraction for classroom scenarios. Yao et al. (2024) proposed DSS-YOLOv7, integrating deformable convolutions and the SE attention mechanism to capture variations in student behavior, achieving 85% mAP@50 on the SCB-Dataset. Zhao and Zhu (2023) designed CBPH-Net, a lightweight detector combining Transformer and bi-directional pyramid networks for real-time classroom behavior recognition. Sheng et al. (2025) improved YOLOv8s through multi-scale feature enhancement strategies to address small target detection challenges in classroom settings. Wang Z. et al. (2024) explored multi-scale deformable transformers for student learning behavior detection, demonstrating the potential of attention-based architectures in educational scenarios. Wang Z. et al. (2025) proposed YOLO-FR, a lightweight detector incorporating feature pyramid shared convolution and RepGhost fusion modules for multi-scale classroom behavior recognition, achieving improved accuracy with reduced computational overhead. Peng et al. (2025) designed YOLO-CBD based on YOLOv10s, introducing BiFormer attention and a novel feature aggregation module to address severe occlusions and scale variations in student behavior detection. Lu et al. (2025) presented YOLO-AMM, which optimizes multi-dimensional features for real-time classroom behavior detection, achieving a favorable balance between detection speed and accuracy. Furthermore, Cheng et al. (2025) proposed SmartLite-YOLO, which achieves an mAP@50 of 87.5% with only 1.6M parameters through lightweight designs including high-frequency enhanced residual blocks, focal modulation, and dynamic upsampling, providing an effective solution for lightweight deployment in classroom behavior detection. However, this method primarily focuses on model compression and small-to-medium target detection, with relatively limited attention to background-interference suppression and multi-directional pose modeling in dense scenes.

Despite these contributions, existing methods still face limitations in simultaneously addressing background interference, multi-directional pose variations, and scale inconsistency. To overcome these limitations, we propose StudentNet, a unified behavior detection framework tailored for the complexities of smart classrooms. Our approach departs from generic improvements by introducing three domain-specific modules designed to address the challenges of clutter, pose variation, and scale inconsistency.

Specifically, StudentNet incorporates three domain-specific modules: (1) a Behavior-Enhanced Fusion Super-Resolution Network (BEF-SRNet) that reconstructs fine-grained features to suppress background interference (2) a Direction-Sensitive Adaptive Convolution (DSAC) module that captures multi-directional pose variations through adaptive receptive fields, and (3) an Adaptive Spatial Feature Fusion (ASFF) strategy that harmonizes cross-scale features for consistent detection across varying target sizes.

Beyond algorithmic improvements, this study also addresses the gap between theoretical research and practical deployment. We further deploy the proposed model on the Core-3399Pro-JD4, a resource-constrained edge computing platform. With INT8 quantization, StudentNet achieves real-time inference speed suitable for live classroom monitoring while maintaining competitive detection accuracy.

In summary, the main contributions of this work are as follows:

(1) We propose a unified behavior detection framework, StudentNet, that effectively reduces false positives and false negatives in crowded classroom environments through the synergy of feature reconstruction, adaptive convolution, and multi-scale fusion.
(2) BEF-SRNet is designed to disentangle behavioral features from cluttered backgrounds, DSAC is introduced to extract direction-sensitive structural cues, and ASFF is employed to maintain scale-invariant feature representations.
(3) Experiments on SCB-Dataset3 show that StudentNet achieves a 6.3% improvement in mAP@50 and a 6.4% increase in F1-score over the YOLOv8n baseline, yielding competitive results among existing methods.
(4) The practical applicability of the proposed method is validated through deployment on the Rockchip RK3399Pro edge platform, where the quantized model achieves 38.4 FPS, meeting the real-time requirements of smart education systems.

## 2 Materials and methods

### 2.1 Overall architecture

Built upon the YOLOv8 baseline, StudentNet introduces three novel components designed to address the specific challenges of classroom behavior detection: the Behavior-Enhanced Fusion Super-Resolution Network (BEF-SRNet) for disentangling background clutter, the Direction-Sensitive Adaptive Convolution (DSAC) module for capturing multi-directional poses, and the Adaptive Spatial Feature Fusion (ASFF) strategy for scale-invariant detection.

### 2.2 Behavior-enhanced fusion super-resolution network

In student behavior recognition tasks, complex classroom environments are often characterized by a high density of students, severe occlusion, and background interference. These factors cause feature aliasing during feature extraction, resulting in high false-recognition and miss rates as well as reduced overall accuracy. Existing super-resolution networks, such as ESPCN (Shi et al., 2016) and SRResNet (Ledig et al., 2017), are primarily designed to improve overall image quality by uniformly reconstructing high-frequency details across the entire spatial domain. However, in classroom behavior detection, the critical information lies in localized behavioral regions such as arm posture and head orientation, rather than in global image quality. Directly applying generic super-resolution methods would indiscriminately enhance both foreground targets and background clutter, failing to improve the discriminability of behavioral features. To address this limitation, we propose a Behavior-Enhanced Fusion Super-Resolution Network (BEF-SRNet), which differs from conventional super-resolution approaches in three aspects. First, the Behavioral Feature Aggregation (BFA) module jointly models local features at multiple scales along with the global context, specifically targeting individual action representation in multi-person crowded scenes. Second, the Behavior-aware Convolutional Feed-forward Network (BCFN) employs partial convolution to selectively process foreground regions while suppressing background noise, rather than treating all spatial locations equally. Third, a region attention mechanism is introduced to emphasize behavior-relevant spatial areas at the semantic level, ensuring that the subsequent PixelShuffle upsampling reconstructs fine-grained details specifically for behavioral regions.

BEF-SRNet consists of multiple stacked behavior fusion blocks (BFBs). Each BFB contains a Behavioral Feature Aggregation (BFA) and a Behavior-aware Convolutional Feed-forward Network (BCFN). Through multi-scale aggregation, the BFA module jointly models local features at different scales and global context in crowded scenes, thereby preserving accurate representations of individual actions when multiple students appear simultaneously. The BCFN module further uses partial convolution to suppress background noise, enabling the network to highlight behavior-related regions and maintain robustness under occlusion and complex environmental conditions.

On this basis, the network introduces a region attention mechanism, which emphasizes key spatial areas and maintains feature consistency at the semantic level. Finally, PixelShuffle upsampling reconstructs the fused features, allowing the high-resolution representation to capture both fine-grained action details and overall semantic consistency, thereby providing more reliable input for subsequent detection and classification. For BEF-SRNet, the feature transformation and final fused output are defined in Equations 1, 2, respectively.


$$F_{b\,f\,a}^{i}=B\,F\,A\,\left(F_{i\,n}^{i}\right),F_{b\,c\,f\,n}^{i}=B\,C\,F\,N\,\left(F_{b\,f\,a}^{i}\right)$$ (1)

$$F_{o\,u\,t}=P\,i\,x\,e\,l\,S\,h\,u\,f\,f\,l\,e\,\left(C\,o\,n\,v_{3=3}\,\left(\sum_{i=1}^{N}\left(F_{b\,c\,f\,n}^{i}+F_{0}\right)\right)\right)$$ (2)

Where *F^i*_*in*_ denotes the input feature of the i-th layer, *F_0_* denotes the shallow feature, and *F*_*out*_ denotes the final fused output. Through this design, BEF-SRNet separates discriminative individual features in dense multi-person scenes, thereby improving the accuracy and robustness of student behavior recognition.

### 2.3 Direction-sensitive adaptive convolution module

Student behavior detection in complex classroom environments faces two major challenges. First, different student behaviors are inherently characterized by distinct spatial orientations. In contrast, “hand-raising” is defined by a prominent vertical arm extension above the head, generating strong vertical structural patterns. Meanwhile, “reading” and “writing” both present a head-down posture, but exhibit subtle differences in arm spatial distribution that also rely on fine-grained directional feature extraction. Second, background regions in classroom scenes also contain rich directional edge information, and these behavior-irrelevant directional features can easily be confused with the behavioral features of foreground targets, increasing the risk of false detections.

The YOLOv8 backbone uses standard 3 × 3 convolutions, where a symmetric kernel W produces the output response at each spatial location, as formulated in Equation 3:


$$y\,\left(i,j\right)=\overset{1}{\underset{m=-1}{\sum }}\overset{1}{\underset{n=-1}{\sum }}W\,\left(m,n\right)\cdot x\,\left(i+m,j+n\right)$$ (3)

where *y*(*i*, *j*) denotes the output feature response at spatial position (*i*, *j*), *W*(*m*, *n*) represents the weight of the convolution kernel at relative offset (*m*, *n*), and *x*(*i* + *m*, *j* + *n*) is the input feature value at the corresponding position. The summation indices m and n both range over {-1, 0, 1}, covering the 3 × 3 spatial neighborhood centered at (*i*, *j*).

This operation applies identical weights across all spatial directions, yielding an isotropic receptive field with equal sensitivity to horizontal, vertical, and diagonal features. Consequently, when two behaviors differ primarily in their dominant orientation, the standard convolution produces similar activation patterns, leading to semantic ambiguity.

Existing studies have attempted to enhance directional feature extraction using asymmetric convolutions. For instance, ACNet decomposes a standard 3 × 3 convolution into three parallel branches (3 × 3, 1 × 3, 3 × 1) and sums their outputs to strengthen feature representation during training (Ding et al., 2019). However, ACNet merges these branches into a single 3 × 3 kernel during inference, thereby losing the explicit directional selectivity.

To overcome these limitations, we propose the Direction-Sensitive Adaptive Convolution (DSAC) module. Unlike ACNet, DSAC maintains four independent directional branches with direction-specific asymmetric padding throughout both training and inference, preserving the explicit modeling capability for orientation-dependent features. Furthermore, DSAC incorporates a channel attention mechanism after branch concatenation to adaptively learn the contribution weights of each directional branch, enabling the network to dynamically emphasize the most discriminative directional information for different behavior classes while suppressing behavior-irrelevant directional responses.

Specifically, DSAC uses asymmetric padding to construct horizontal 1 × 3 and vertical 3 × 1 convolution kernels, enabling directional feature extraction from different regions of the input. For the input tensor X with dimensions (h, w, c), where h, w, and c denote height, width, and channel number, respectively, batch normalization and the SiLU activation function are applied after each convolution to stabilize training and accelerate convergence. The first layer of DSAC performs parallel convolution across four directional branches, and the specific operations are defined in Equation 4:


$$X_{1}^{\left(h^{{}^{\prime }},w^{{}^{\prime }},c^{{}^{\prime }}\right)}=S\,i\,L\,U\,\left(B\,N\,\left(X_{P\,\left(1,0,0,3\right)}^{\left(h_{1},w_{1},c_{1}\right)}\,⊗W_{1}^{\left(1,3,c^{{}^{\prime }}\right)}\right)\right),$$ (4)

$$X_{2}^{\left(h^{{}^{\prime }},w^{{}^{\prime }},c^{{}^{\prime }}\right)}=S\,i\,L\,U\,\left(B\,N\,\left(X_{P\,\left(0,3,0,1\right)}^{\left(h_{1},w_{1},c_{1}\right)}\,⊗W_{2}^{\left(3,1,c^{{}^{\prime }}\right)}\right)\right),$$

$$X_{3}^{\left(h^{{}^{\prime }},w^{{}^{\prime }},c^{{}^{\prime }}\right)}=S\,i\,L\,U\,\left(B\,N\,\left(X_{P\,\left(0,1,3,0\right)}^{\left(h_{1},w_{1},c_{1}\right)}\,⊗W_{3}^{\left(1,3,c^{{}^{\prime }}\right)}\right)\right),$$

$$X_{4}^{\left(h^{{}^{\prime }},w^{{}^{\prime }},c^{{}^{\prime }}\right)}=S\,i\,L\,U\,\left(B\,N\,\left(X_{P\,\left(3,0,1,0\right)}^{\left(h_{1},w_{1},c_{1}\right)}\,⊗W_{4}^{\left(3,1,c^{{}^{\prime }}\right)}\right)\right)$$

where ⊗ denotes the convolution operation, and W1(1, 3,c′) denotes a 1 × 3 convolution kernel with *c*′ output channels. The padding parameter P(1,0,0,3) specifies the number of padding pixels in each direction, corresponding to 1 pixel on the left, 0 pixels on the right, 0 pixels on the top, and 3 pixels on the bottom.

Following the first layer of parallel convolution, the dimensions (height, width, and number of channels) of the output feature map are calculated using Equation 5:


$$h^{{}^{\prime }}=\frac{h_{1}}{s}+1,w^{{}^{\prime }}=\frac{w_{1}}{s}+1,c^{{}^{\prime }}=\frac{c_{2}}{4}\;$$ (5)

where *c*_2_ denotes the number of channels in the final output feature map of the DSAC module, and s denotes the convolution stride. The outputs of the first-layer parallel convolutions are concatenated using [Cat(.,.)], as shown in Equation 6:


X(h′,w′,4c′)′=C⁢a⁢t⁢(X1(h′,w′,c′),…,X4(h′,w′,c′)) (6)

Then, global average pooling compresses the spatial dimensions of the feature map from h × w × 4c to 1 × 1 × 4c, generating a channel descriptor vector that captures global contextual information. To improve the generalization ability and reduce model complexity, activation functions are employed to assign weights to feature channels based on their inter-channel correlations, thereby highlighting important channels. A bottleneck structure consisting of two fully connected layers is used to capture inter-channel dependencies. The first fully connected layer performs dimensionality reduction. After applying the ReLU activation function, the second fully connected layer restores the original dimensionality and multiplies the learned activation values by the original features. Finally, the importance of each channel is predicted through the Sigmoid function, and this operation is formulated in Equation 7:


$$s=F_{e\,x}\,\left(Z,V\right)=\sigma \,\left(V_{2}\,\delta \,\left(V_{1}\,Z\right)\right)$$ (7)

where s represents the channel-wise weight, Z denotes the global feature descriptor obtained after compression, *F*_*ex*_(⋅) denotes the excitation function, and V denotes the fully connected layer parameter.

The output $\tilde{X}$ is obtained by applying the weight *s_c_* of the inter-channel dependency to the original feature map *u_c_* for scaling, as expressed in Equation 8:


$$\tilde{X}=F_{s\,c\,a\,l\,e}\,\left(u_{c},s_{c}\right)=u_{c}\,\text{×}\,s_{c}\;$$ (8)

where *F*_*scale*_ denotes channel-wise multiplication between the weight vector *s_c_* and the feature map *u_c_* and *u_c_* denotes the c-th channel of the input feature map.

Finally, the concatenated tensor is processed by a convolution kernel W without padding. As shown in Equation 9, the height and width of the output feature map are adjusted to preset values *h*_*2*_ and *w*_*2*_, making DSAC interchangeable with a standard convolutional layer while enabling channel attention to evaluate the contributions of different convolution directions. The final output Y is computed using Equation 10:


$$h_{2}=h^{{}^{\prime }}-1=\frac{h_{1}}{s},w_{2}=w^{{}^{\prime }}-1=\frac{w_{1}}{s}\;$$ (9)

Y=S⁢i⁢L⁢U⁢(B⁢N⁢(X(h′,w′,4c′)′⁢⊗W)) (10)

### 2.4 Adaptive spatial feature fusion strategy

The YOLOv8 architecture employs an enhanced PAN-FPN configuration within its neck component, which facilitates efficient feature aggregation during the upsampling phase. Nevertheless, the integration of multi-scale features may introduce semantic conflicts and feature inconsistencies across different pyramid levels. This challenge is further exacerbated in student behavior recognition tasks by factors including cluttered classroom backgrounds and substantial scale variations among target instances. The Adaptive Spatial Feature Fusion (ASFF) mechanism was originally proposed by Liu et al. (2019). for general object detection. In this work, ASFF is incorporated into the detection head with targeted adaptations for the student behavior detection task. Standard feature pyramid fusion methods, such as PAN-FPN, rely on fixed concatenation or addition operations, which may introduce semantic conflicts among features from different scales. ASFF addresses this issue through learnable spatial weights that adaptively determine the contribution of each scale at every spatial position, which is particularly beneficial for maintaining consistent detection performance across the extreme scale variations inherent in classroom environments. Through hierarchical feature integration, three ASFF modules, designated as ASFF-1, ASFF-2, and ASFF-3, are constructed to process features at their respective scales.

The ASFF mechanism achieves effective multi-scale feature integration through a two-stage process consisting of feature-scale alignment and learnable weight-based fusion. To ensure spatial and channel consistency across pyramid levels, the strategy employs scale-specific transformations. For upsampling operations, 1 × 1 convolutions are first applied to compress the feature channels, aligning them with the target layer’s channel dimensionality, followed by interpolation to increase the spatial resolution of the feature maps. Conversely, downsampling at a 1/2 ratio utilizes 3 × 3 convolutions with a stride of 2 to reduce the feature map dimensions by half while simultaneously adjusting the channel count. For downsampling at a 1/4 ratio, an additional max pooling layer with a stride of 2 is incorporated into the 1/2 ratio downsampling pipeline, also employing 3 × 3 convolutions. This parameterized mechanism ensures that features from different scales are transformed into a unified dimensional space, facilitating seamless integration.

The aggregated feature representation at each spatial location is computed through a weighted combination of scale-normalized feature maps, where each feature undergoes element-wise multiplication with its corresponding learnable weight matrix, followed by summation. This process is formally expressed in Equation 11:


$$y_{i\,j}^{l}=\alpha_{i\,j}^{l}\cdot x_{i\,j}^{1\to l}+\beta_{i\,j}^{l}\cdot x_{i\,j}^{2\to l}+\gamma_{i\,j}^{l}\cdot x_{i\,j}^{3\to l}$$ (11)

where $x_{i\,j}^{n\to l}$ denotes the feature vector transformed from layer *n* to layer *l* at spatial position (*i*, *j*). The parameters $\alpha_{i\,j}^{l},\beta_{i\,j}^{l}\,\mathrm{and}\,\gamma_{i\,j}^{l}\in \left[0,1\right]$constitute learnable fusion weights that determine the contribution of each scale-specific feature at position (*i*, *j*) on layer *l*. These weights are constrained to satisfy the normalization condition shown in Equations 12, 13:


$$\alpha_{i\,j}^{l}=\frac{e^{\lambda_{a_{i,j}}^{l}}}{e^{\lambda_{a_{i,j}}^{l}}+e^{\lambda_{\beta_{i,j}}^{l}}+e^{\lambda_{\gamma_{i,j}}^{l}}}\;$$ (12)

$$\alpha_{i\,j}^{l}+\beta_{i\,j}^{l}+\gamma_{i\,j}^{l}=1$$ (13)

In these equations, the weight parameters $\lambda_{\alpha_{i,j}}^{l},\lambda_{\beta_{i,j}}^{l},\lambda_{\gamma_{i,j}}^{l}$are derived from the scale-aligned feature layers *x*^1→*l*^, *x*^2→*l*^, *x*^3→*l*^through 1 × 1 convolutional operations. Through this adaptive weighting scheme, ASFF strengthens the semantic representation of student behavior across all feature pyramid levels, enabling more effective cross-scale feature fusion and enhancing the discriminative capacity of behavior detection.

## 3 Results and discussion

### 3.1 Datasets and experimental setup

**FIGURE 1:**
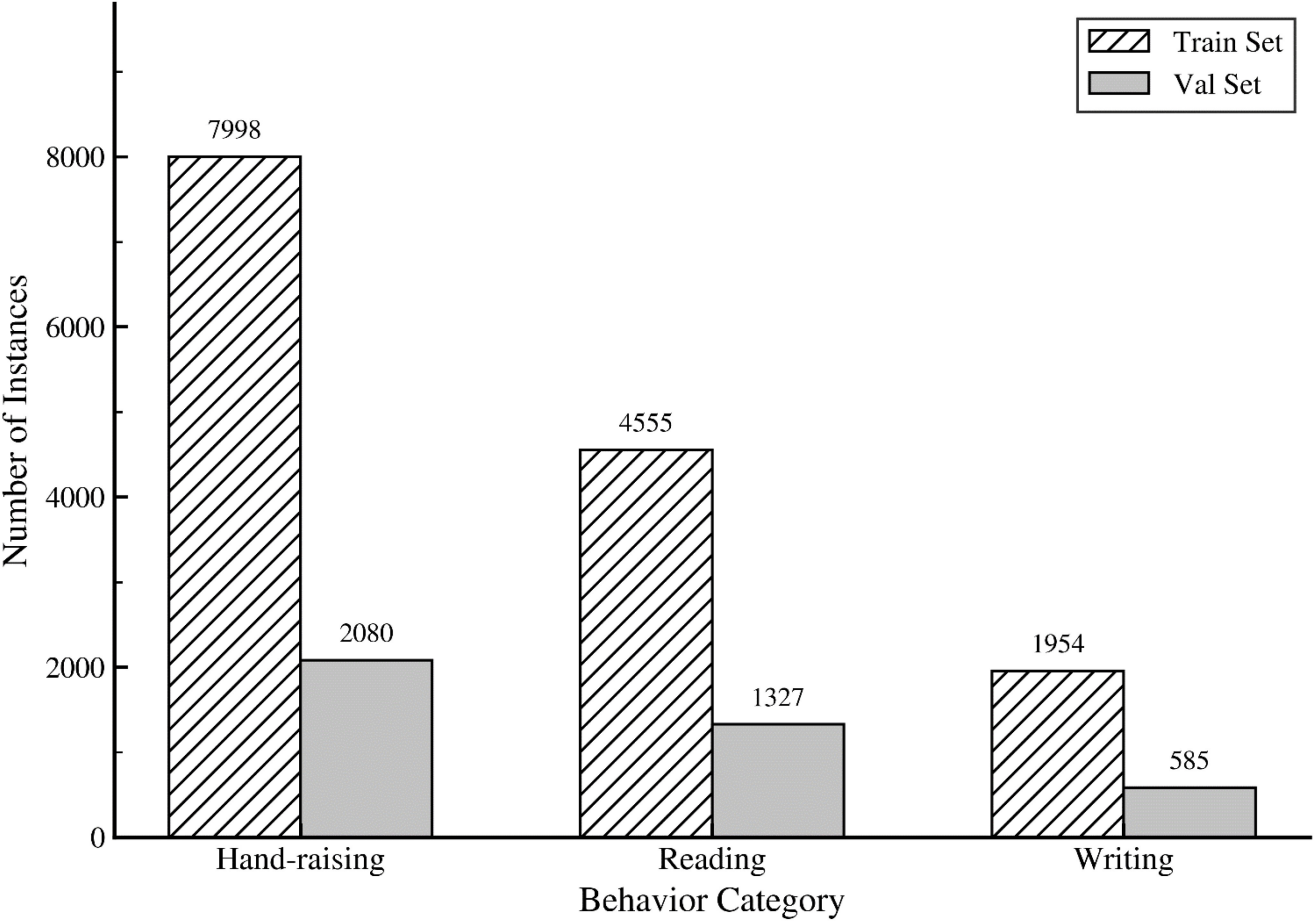
Distribution of annotated instances across behavior categories in the training and validation sets of SCB-Dataset3-S.

We utilized the SCB-Dataset3-S (Yang and Wang, 2023), a subset of the SCB-Dataset3 benchmark specifically curated for complex educational scenarios. The dataset categorizes student behaviors into three classes: hand-raising, reading, and writing. The dataset was divided into a training set containing 3,418 images with 14,507 annotated instances and a validation set containing 848 images with 3,992 annotated instances, totaling 4,266 images with 18,499 annotated instances. As shown in Figure 1, the class-wise distribution exhibits a notable imbalance, with hand-raising accounting for 54.5% of the total instances, followed by reading at 31.8% and writing at 13.7%. These factors, combined with the inherent class imbalance, collectively pose significant challenges for detection algorithms.

All experiments were conducted on a workstation equipped with an Intel Core i9-13900K CPU and an NVIDIA RTX 4090 GPU. The models were implemented in Python 3.9 with PyTorch 2.1.0 and the Ultralytics YOLOv8 framework. Training was performed for 200 epochs using the SGD optimizer with a momentum of 0.937 and a weight decay of 0.0005. An initial learning rate of 0.01 was employed, with a cosine annealing schedule for decay. The batch size was set to 16.

### 3.2 Evaluation metrics

To evaluate the detection performance of the proposed algorithm and the effectiveness of the improvements, we used precision, recall, average precision, and mean average precision as evaluation metrics. These metrics are defined in Equations 14–17:


$$P=\frac{T\,P}{T\,P+F\,P}$$ (14)

$$R=\frac{T\,P}{T\,P+F\,N}$$ (15)

$$A\,P=\int_{0}^{1}P\,\left(R\right)\,dR$$ (16)

$$m\,A\,P=\frac{\sum_{0}^{1}A\,P_{1}}{n}$$ (17)

where TP denotes the number of correctly identified positive samples, FP denotes the number of negative samples incorrectly identified as positive, FN denotes the number of positive samples incorrectly identified as negative, and n denotes the number of detection categories. AP represents the area under the precision–recall curve, whereas mAP denotes the mean AP across all categories.

### 3.3 Ablation experiments

**FIGURE 2:**
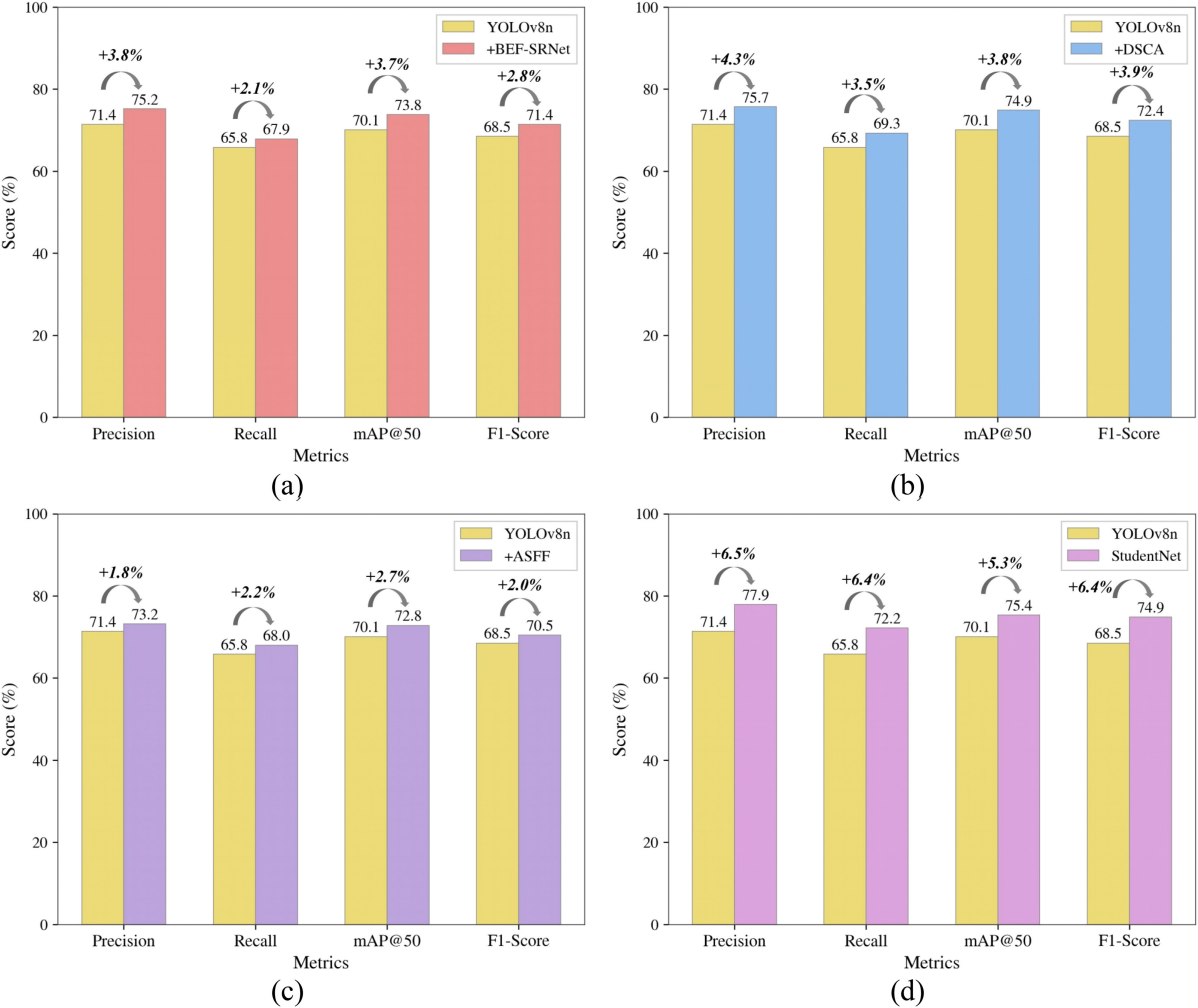
Performance comparison of YOLOv8n with individual module integration. **(a)** YOLOv8n + BEF-SRNet. **(b)** YOLOv8n + DSAC. **(c)** YOLOv8n + ASFF. **(d)** YOLOv8n + All modules. The gray and colored bars represent the YOLOv8n baseline and the enhanced model, respectively.

To verify the effectiveness of each key module in StudentNet and its contribution to overall performance, we conducted ablation experiments. The core of StudentNet consists of three key modules: the Behavior-Enhanced Fusion Super-Resolution Network (BEF-SRNet), the Direction-Sensitive Adaptive Convolution module (DSAC), and the Adaptive Spatial Feature Fusion strategy (ASFF). Based on the YOLOv8n baseline, the three modules were embedded into the network either individually or in different combinations. Each model configuration was trained and evaluated under the same experimental settings, and key performance indicators, including precision and recall, were recorded. The specific experimental results are shown in Figure 2 and Table 1.

**TABLE 1:** Results of ablation experiment.

BEF-SRNet	DSAC	ASFF	Precision%	Recall%	mAP@50%	F1-Score%	Params	GFLOPs
–	–	–	71.4	65.8	70.1	68.5	3.0M	8.1
√	–	–	75.2	67.9	73.8	71.4	4.5M	10.9
–	√	–	75.7	69.3	74.9	72.4	4.7M	10.3
–	–	√	73.2	68.0	72.8	70.5	3.8M	9.6
√	√	–	77.3	70.0	75.7	73.5	5.6M	12.5
√	–	√	75.4	68.6	74.2	71.8	5.2M	11.7
–	√	√	76.1	70.7	75.4	73.3	5.3M	11.4
√	√	√	77.9	72.2	76.4	74.9	6.1M	13.2

When BEF-SRNet was used alone, its multi-scale feature fusion and contextual attention mechanism effectively disentangled student behavior regions from complex classroom backgrounds. Meanwhile, PixelShuffle upsampling preserved behavioral details, reducing false detections caused by background interference and missed detections caused by detail loss, thereby enhancing the discriminability of behavioral features. Precision, recall, mAP@50, and F1-score improved by 3.8, 2.1, 3.7, and 2.9 percentage points, respectively, while the number of parameters increased to 4.5M and the computational cost reached 10.9 GFLOPs. When DSAC was used alone, mAP@50 and F1-score increased by 4.8 and 3.9 percentage points, respectively, while the number of parameters increased to 4.7M and the computational cost reached 10.3 GFLOPs. Owing to its four asymmetric-padding branches and directional convolution kernels, DSAC can capture students’ sitting postures and multi-directional pose features under varying camera angles, addressing the limitation of the fixed receptive field in traditional 3 × 3 convolutions. The ASFF module alleviates information conflicts in YOLOv8n during multi-scale feature fusion by using feature-scale normalization and adaptive weight fusion, thereby assigning dynamic weights to features at different scales and improving robustness to multi-scale behaviors. As a relatively lightweight component, ASFF only increases the parameters to 3.8M with a computational cost of 9.6 GFLOPs. The final row reports the complete StudentNet model, which achieves the best performance, with improvements of 6.5, 6.4, 6.3, and 6.4 percentage points in precision, recall, mAP@50, and F1-score, respectively. The full model comprises 6.1M parameters and 13.2 GFLOPs, striking a favorable balance between detection accuracy and computational overhead.

### 3.4 Comparison with state-of-the-art methods

To comprehensively evaluate the detection capability of StudentNet, this section compares it with several advanced object detection methods on SCB-Dataset3, including RT-DETR (Zhao et al., 2024), SSD (Liu et al., 2016), YOLOv7-tiny (Wang et al., 2023), YOLOv8n, YOLOv10n (Wang A. et al., 2024), Hyper-YOLO (Feng et al., 2024), and Mamba-YOLO (Wang Z. et al., 2025). The quantitative results are summarized in Table 2.

**TABLE 2:** Benchmarking StudentNet against state-of-the-art object detection methods on SCB-dataset3.

Model	F1-Score%	mAP@50%	mAP@50:95%	Params	Model size (MB)	FPS	Latency (ms)
rtdetr-l	68.9	71.6	54.7	32.0M	64.3	34	29.4
SSD	59.7	59.3	34.6	24.8M	49.8	45	22.2
YOLOv7-tiny	68.3	70.0	52.5	6.0M	12.3	135	7.4
YOLOv8n	68.5	70.1	53.4	3.0M	6.2	223	4.5
YOLOv10n	67.8	69.7	52.6	2.3M	5.1	271	3.7
Hyper-YOLO-N	68.8	71.6	55.5	4.0M	8.4	202	5.0
Mamba-YOLO-T	69.7	73.0	55.2	6.1M	12.6	143	7.0
StudentNet	74.9	76.4	58.5	6.1M	12.5	129	7.8

StudentNet achieves the highest detection accuracy among all compared methods, with an F1-score of 74.9%, mAP@50 of 76.4%, and mAP@50:95 of 58.5%. Compared with the YOLOv8n baseline, StudentNet improves F1-score, mAP@50, and mAP@50:95 by 6.4, 6.3, and 5.1 percentage points, respectively. This indicates that StudentNet improves not only coarse localization but also precise bounding-box regression performance.

The RT-DETR-l model achieves an F1-score of 68.9% and an mAP@50 of 71.6% with a parameter count of 32.0M. StudentNet surpasses RT-DETR-l by 6.0% in F1-score and 4.8% in mAP@50, while having only 6.1M parameters. The SSD detector performs the worst among all compared methods, with an F1-score of 59.7% and an mAP@50 of 59.3%, lagging behind StudentNet by 15.2 and 17.1 percentage points, respectively. The limited ability of SSD to handle severe scale variations and dense occlusions in classroom environments results in insufficient detection accuracy.

YOLOv10n achieves the highest inference speed among the YOLO-series models at 271 FPS, but its F1-score is only 67.8% and its mAP@50 is 69.7%. StudentNet outperforms YOLOv10n by 7.1% in F1-score and 6.7% in mAP@50. Although YOLOv10n employs an NMS-free design to accelerate inference, its detection accuracy in complex classroom scenes is limited due to the lack of dedicated modules for background suppression and multi-directional feature extraction. Mamba-YOLO-T, which uses a state-space model to enhance feature representation, achieves an F1-score of 69.7% and an mAP@50 of 73.0% with 6.1M parameters. With a comparable parameter count, StudentNet still surpasses Mamba-YOLO-T by 5.2% in F1-score and 3.4% in mAP@50.

The performance advantage of StudentNet is attributed to the effective synergy of its three proposed modules. BEF-SRNet separates behavioral features from the cluttered classroom background through multi-scale feature reconstruction, reducing false detections caused by environmental interference. DSAC employs asymmetric convolution kernels with directional padding to capture multi-directional pose features, achieving robust discrimination between visually similar behaviors like reading and writing. ASFF harmonizes features from different pyramid levels using learnable spatial weights, ensuring consistent detection performance for both large-scale targets in the front rows and small-scale targets in the back rows.

In contrast, the proposed method detects student behaviors more effectively and achieves more robust performance in complex classroom scenes.

**FIGURE 3:**
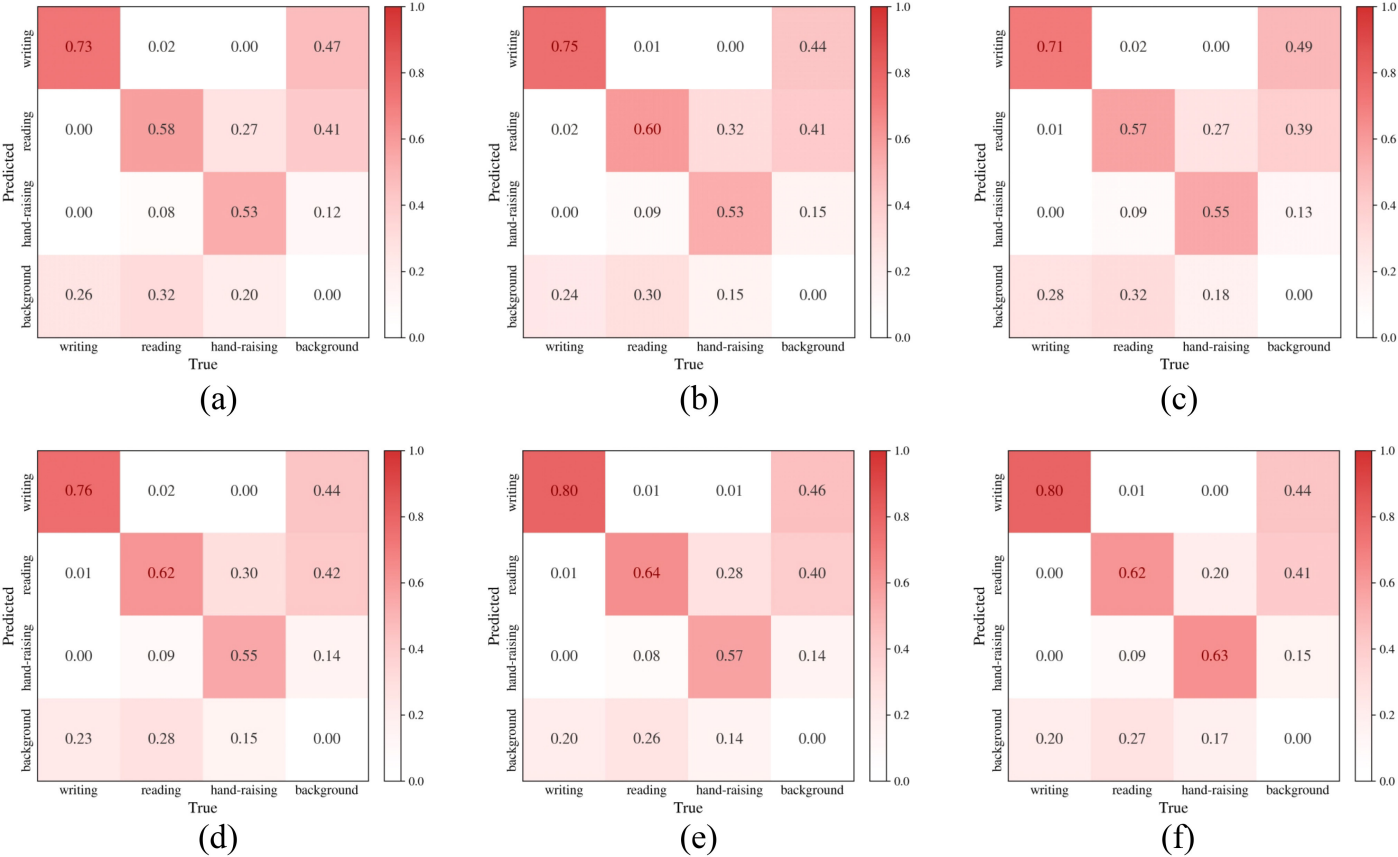
Comparison of confusion matrices for different models on the student behavior classification task. **(a)** YOLOv7-tiny. **(b)** YOLOv8n. **(c)** YOLOv10n. **(d)** Hyper-YOLO-N. **(e)** Mamba-YOLO-T. **(f)** StudentNet.

To further analyze the detection performance of StudentNet across different behavior classes, the confusion matrices are presented in Figure 3. The results indicate that the model achieves the highest recognition accuracy for the “writing” class, while the performance for “reading” and “hand-raising” is comparable. The “writing” behavior exhibits relatively stable postural features, thus leading to higher classification accuracy. The recognition rate for “hand-raising” is relatively lower due to variations in arm position and occlusion in crowded scenes. This is primarily because “reading” and “hand-raising” can appear visually similar when observed from certain angles. Nevertheless, thanks to the ability of the DSAC module to capture directional features, our model still maintains a lower misclassification rate compared to the baseline.

**FIGURE 4:**
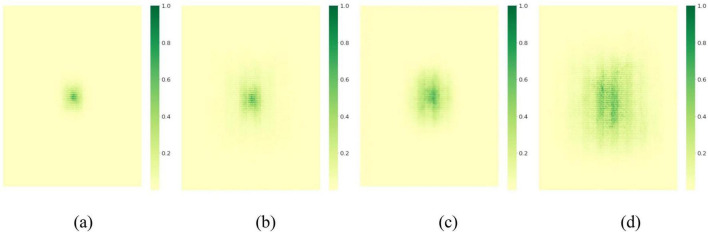
Effective receptive field (ERF) comparison of YOLOv8 and StudentNet. **(a,b)** the ERF of the first detection head layer of YOLOv8 and StudentNet, respectively. **(c,d)** The ERF of the last backbone layer of YOLOv8 and StudentNet, respectively. Green regions indicate a higher contribution to the feature response.

To investigate the structural characteristics of the networks, we analyzed the effective receptive fields (ERFs) of YOLOv8 and StudentNet. The corresponding findings are presented in Figure 4, which demonstrates that StudentNet achieves a larger effective receptive field than YOLOv8.

Specifically, the receptive field area shown in Figure 4b is larger than that in Figure 4a. This difference is particularly notable along the edge regions, where StudentNet maintains a wider detection scope than YOLOv8. Additionally, the effective receptive field area in Figure 4d is larger than that in Figure 4c. These observations further demonstrate the enhanced feature representation capability of StudentNet.

The experiment used a single image as the input sample and focused on visualizing the feature maps of layers 3, 5, and 7 of the two models. The results show that as the network depth increases, the spatial information contained in the feature maps becomes progressively clearer. In comparison, StudentNet not only outperforms YOLOv8n in extracting multi-class foreground target features but also consistently maintains high sensitivity to targets of different scales.

## 4 Algorithm deployment

**FIGURE 5:**
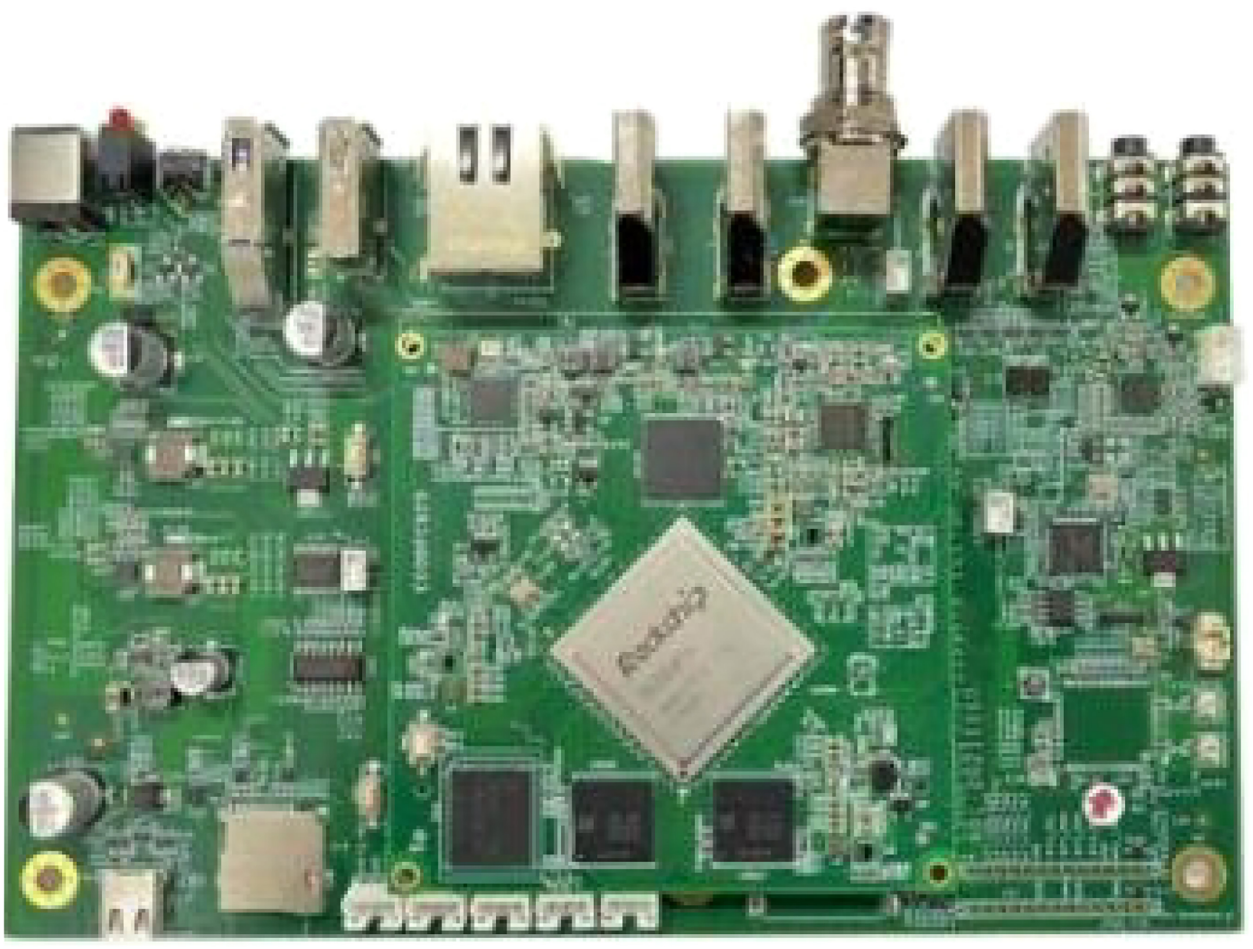
The Core-3399Pro-JD4 edge computing platform used for algorithm deployment.

The hardware platform used in this experiment is the Core-3399Pro-JD4 development board, which is a high-performance embedded platform specifically designed for edge intelligent computing scenarios. Its core is equipped with the Rockchip RK3399Pro main control chip, which adopts a heterogeneous computing architecture integrating dual Cortex-A72 high-performance cores and quad Cortex-A53 energy-efficient cores, effectively controlling the overall power consumption while ensuring adequate computing performance. The development board is also equipped with a dedicated neural network processing unit (NPU) that is optimized for 8-bit/16-bit integer operations, and the NPU provides efficient computing power while consuming only approximately 1% of the power of GPUs at the same performance level. This high energy-efficiency ratio provides significant advantages in edge computing scenarios, making it especially suitable for embedded deployments with strict power and form-factor constraints. The development board is shown in Figure 5.

**FIGURE 6:**
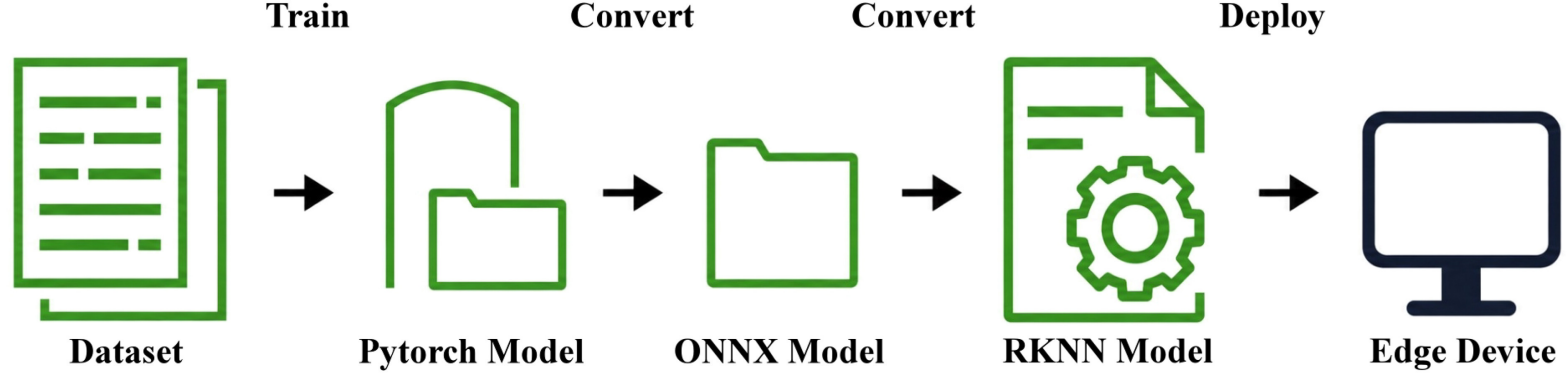
Workflow for model quantization and deployment on the Core-3399Pro-JD4 edge platform.

In the practical deployment of deep learning models, traditional direct deployment methods are often limited by framework compatibility and runtime performance bottlenecks. To reduce dependence on specific frameworks, we adopted an efficient model deployment strategy. First, the YOLOv8n and StudentNet models were trained using the PyTorch framework to obtain the optimal model weights. To ensure cross-platform compatibility and stability, the model weights were exported to the Open Neural Network Exchange (ONNX) format with static batch processing. This setting fixes all tensor dimensions during inference, ensuring consistent inference behavior and improving compatibility across diverse deployment scenarios. To enable efficient deployment on the RK3399Pro platform, the RKNN-Toolkit (version 1.7.5) was employed for model conversion and optimization. The Core-3399Pro-JD4 development board runs Ubuntu 18.04 with NPU driver version 1.7.5. This toolkit is specifically designed for Rockchip’s NPU and supports various optimization techniques including operator fusion, memory optimization, and quantization calibration. The RKNN-Toolkit converts the ONNX model into the RKNN format, which is optimized for the neural processing unit (NPU) of the RK3399Pro. To achieve higher inference speed without significant accuracy degradation, INT8 quantization is applied to the StudentNet model during the conversion process. With this deployment pipeline, inference requires only the optimized model weights and no longer depends on the original training framework. This strategy reduces inference time and enables efficient edge deployment of the deep learning model. The deployment process is illustrated in Figure 6.

During the model export phase, the trained StudentNet model was exported from the PyTorch framework to the ONNX format with the opset version set to 12. The input size was fixed at 640 × 640, and static batch processing (batch size = 1) was adopted to ensure deterministic and consistent inference dimensions. In the RKNN model conversion phase, the RKNN-Toolkit was employed to convert and optimize the ONNX model. For the INT8 quantization calibration dataset, 200 representative images were randomly selected from the training set of SCB-Dataset3, covering diverse lighting conditions, student densities, and behavior categories, to ensure that the quantization process adequately captures the statistical characteristics of the feature distributions. The INT8 quantization precision-loss rate was calculated by comparing mAP@50 before and after quantization on the validation set, as follows: Precision Loss Rate = (mAP_FP32 - mAP_INT8)/mAP_FP32 × 100%. The experimental results showed that the mAP@50 of the quantized model decreased from 76.4 to 71.7%, corresponding to a precision-loss rate of approximately 6.2%, which is within an acceptable range. To ensure measurement reliability, all inference speed evaluations on the edge platform were preceded by 50 warm-up iterations to stabilize hardware performance, and the reported FPS values were averaged over 200 consecutive inference iterations.

**FIGURE 7:**
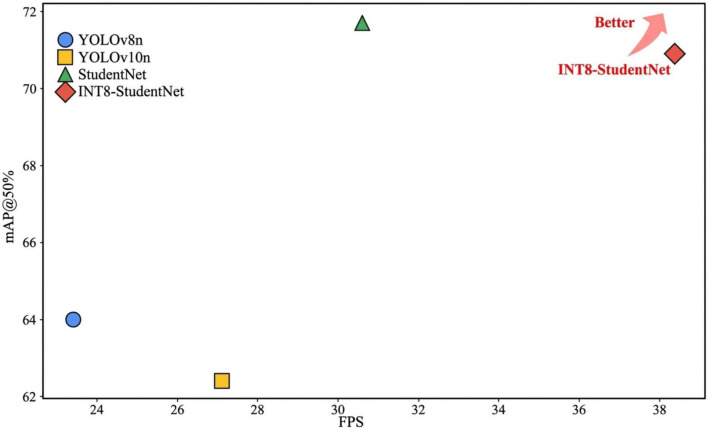
Inference performance analysis on the Core-3399Pro-JD4 edge platform.

Figure 7 presents a comparison of inference performance among different models on the Core-3399Pro-JD4 platform. As observed from the scatter plot, StudentNet and INT8-StudentNet are positioned in the upper-right region, indicating that they outperform the compared YOLO-series models in both accuracy and speed. Specifically, StudentNet achieves the highest detection accuracy, with mAP@50 reaching 71.7%. Meanwhile, the INT8-quantized model significantly boosts inference speed to 38.4 FPS while maintaining relatively high accuracy. Overall, StudentNet achieves strong real-time performance while maintaining high accuracy, demonstrating its practical advantages in edge deployment scenarios.

## 5 Conclusion

In this study, we proposed StudentNet, a novel student behavior detection framework designed for complex classroom environments. The framework integrates three key modules: BEF-SRNet for multi-scale super-resolution feature fusion to suppress background interference, DSAC for multi-directional adaptive feature extraction to capture pose variations, and ASFF for scale-consistent spatial feature integration to address target size inconsistency. Experimental results on SCB-Dataset3 demonstrate that StudentNet achieves considerable improvements over several representative detectors such as YOLOv8n and YOLOv10n in terms of precision, recall, and mAP. Ablation studies further confirm the effectiveness of each proposed module. To further validate its practical applicability, StudentNet was successfully deployed on the Core-3399Pro-JD4 edge computing platform. After INT8 quantization, the model maintained competitive detection accuracy while achieving 38.4 FPS, highlighting an effective balance between accuracy and real-time performance.

Nevertheless, this work has certain limitations. The current evaluation is limited to a single dataset with three behavior categories, and its generalizability to more diverse classroom scenarios and richer behavior taxonomies remains to be investigated. Additionally, the increased model complexity compared with the baseline may limit deployment on more severely resource-constrained platforms. Furthermore, the current deployment experiments primarily focused on validating inference throughput and quantization accuracy, but did not incorporate a complete end-to-end latency profiling pipeline. In future work, we will extend the framework to support a broader range of student behaviors, explore knowledge distillation and more efficient quantization strategies to further reduce the model footprint, and conduct comprehensive end-to-end latency benchmarking on edge platforms, covering image acquisition, inference, and result output, to provide a more thorough evaluation of real-time deployment performance.

## Data Availability

The original contributions presented in this study are included in the article/supplementary material, further inquiries can be directed to the corresponding author.

## References

[B1] Buono P. De Carolis B. D’Errico F. Macchiarulo N. Palestra G. (2023). Assessing student engagement from facial behavior in on-line learning. *Multimed. Tools Appl*. 82 12859–12877. 10.1007/s11042-022-14048-8 36313482 PMC9589763

[B2] Chen H. Zhou G. Jiang H. (2023). Student behavior detection in the classroom based on improved YOLOv8. *Sensors* 23:8385. 10.3390/s23208385 37896479 PMC10611206

[B3] Cheng F. Zhang H. Zhang T. (2025). SmartLite-YOLO: a smart classroom lightweight YOLO for student behavior recognition. *J. Electron. Imaging* 34:053034. 10.1117/1.JEI.34.5.053034

[B4] Dimitriadou E. Lanitis A. (2023). A critical evaluation, challenges, and future perspectives of using artificial intelligence and emerging technologies in smart classrooms. *Smart Learn. Environ*. 10:12. 10.1186/s40561-023-00231-3 40478141 PMC9900563

[B5] Ding X. Guo Y. Ding G. Han J. (2019). “Acnet: strengthening the kernel skeletons for powerful cnn via asymmetric convolution blocks,” in *Proceedings of the IEEE/CVF International Conference on Computer Vision*, (Seoul), 1911–1920.

[B6] Feng Y. Huang J. Du S. Ying S. Yong J. H. Li Y. et al. (2024). Hyper-YOLO: when visual object detection meets hypergraph computation. *IEEE Trans. Pattern Anal. Mach. Intell*. 47 2388–2401. 10.1109/TPAMI.2024.3524377 40030788

[B7] Han L. Ma X. Dai M. Bai L. (2025). A WAD-YOLOv8-based method for classroom student behavior detection. *Sci. Rep*. 15:9655. 10.1038/s41598-025-87661-w 40113824 PMC11926185

[B8] Jia Q. He J. (2024). Student behavior recognition in classroom based on deep learning. *Appl. Sci.* 14:7981. 10.3390/app14177981

[B9] Ledig C. Theis L. Huszár F. Caballero J. Cunningham A. Acosta A. et al. (2017). “Photo-realistic single image super-resolution using a generative adversarial network,” in *Proceedings of the IEEE Conference on Computer Vision and Pattern Recognition*, (Vancouver, BC), 4681–4690.

[B10] Li Y. Qi X. Saudagar A. K. Badshah A. M. Muhammad K. Liu S. (2023). Student behavior recognition for interaction detection in the classroom environment. *Image Vis. Comput.* 136:104726. 10.1016/j.imavis.2023.104726

[B11] Liu S. Huang D. Wang Y. (2019). Learning spatial fusion for single-shot object detection. *arXiv [Preprint].* 10.48550/arXiv.1911.09516

[B12] Liu W. Anguelov D. Erhan D. Szegedy C. Reed S. Fu C. Y. et al. (2016). “Ssd: single shot multibox detector,” in *Proceedings of the European Conference on Computer Vision*, (Cham: Springer International Publishing).

[B13] Lu W. Liu S. Ding B. Chen P. Lu F. (2025). Student behavior detection model based on multilevel residual networks and hybrid attention mechanisms. *Neurocomputing* 635:129965. 10.1016/j.neucom.2025.129965

[B14] Ngoc Anh B. Tung Son N. Truong Lam P. Phuong Chi L. Huu Tuan N. Cong Dat N. et al. (2019). A computer-vision based application for student behavior monitoring in classroom. *Appl. Sci.* 9:4729. 10.3390/app9224729

[B15] Peng S. Zhang X. Zhou L. Wang P. (2025). YOLO-CBD: classroom behavior detection method based on behavior feature extraction and aggregation. *Sensors* 25:3073. 10.3390/s25103073 40431867 PMC12115732

[B16] Ragab M. G. Abdulkadir S. J. Aziz N. Al-Tashi Q. Alyousifi Y. Alhussian H. et al. (2024). A comprehensive systematic review of YOLO for medical object detection (2018 to 2023). *IEEE Access* 12 57815–57836. 10.1109/ACCESS.2024.3386826

[B17] Sheng X. Li S. Chan S. (2025). Real-time classroom student behavior detection based on improved YOLOv8s. *Sci. Rep*. 15:14470. 10.1038/s41598-025-99243-x 40281063 PMC12032004

[B18] Shi W. Caballero J. Huszár F. Totz J. Aitken A. P. Bishop R. et al. (2016). “Real-time single image and video super-resolution using an efficient sub-pixel convolutional neural network,” in *Proceedings of the IEEE Conference on Computer Vision and Pattern Recognition*, (Las Vegas, NV), 1874–1883.

[B19] Trabelsi Z. Alnajjar F. Parambil M. M. A. Gochoo M. Ali L. (2023). Real-time attention monitoring system for classroom: a deep learning approach for student’s behavior recognition. *Big Data Cognit. Comput.* 7:48. 10.3390/bdcc7010048

[B20] Wang A. Chen H. Liu L. Chen K. Lin Z. Han J. et al. (2024). “Yolov10: real-time end-to-end object detection,” in *Proceedings of the 38th International Conference on Advances in Neural Information Processing Systems*, (Vancouver, BC), 107984–108011.

[B21] Wang C. Mohamed A. S. Yang X. Zhang H. Li X. Halim Mohd Noor M. (2025). Enhancing classroom behavior recognition with lightweight multi-scale feature fusion. *Comput. Mater. Continua* 85 855–874. 10.32604/cmc.2025.066343

[B22] Wang C.-Y. Bochkovskiy A. Mark Liao H.-Y. (2023). “YOLOv7: trainable bag-of-freebies sets new state-of-the-art for real-time object detectors,” in *Proceedings of the IEEE/CVF Conference on Computer Vision and Pattern Recognition*, (Vancouver, BC).

[B23] Wang Z. Li C. Xu H. Zhu X. Li H. (2025). “Mamba yolo: a simple baseline for object detection with state space model,” in *Proceedings of the AAAI Conference on Artificial Intelligence*, (Philadelphia, PA).

[B24] Wang Z. Wang M. Zeng C. Li L. (2024). Multi-scale deformable transformers for student learning behavior detection in smart classroom. *arXiv [Preprint].* 10.48550/arXiv.2410.07834

[B25] Yang B. Yao Z. Lu H. Zhou Y. Xu J. (2020). In-classroom learning analytics based on student behavior, topic and teaching characteristic mining. *Pattern Recogn. Lett.* 129 224–231. 10.1016/j.patrec.2019.11.023

[B26] Yang F. Wang T. (2023). Scb-dataset3: a benchmark for detecting student classroom behavior. *arXiv [Preprint].* 10.48550/arXiv.2310.02522

[B27] Yao F. Chen X. Jiang Y. Jia W. (2024). “DSS-YOLOv7: an enhanced YOLOv7-based algorithm for classroom behavior detection,” in *Proceedings of the 2024 7th International Conference on Machine Learning and Machine Intelligence*, (Osaka), 281–289.

[B28] Yin C. C. Sun Y. Li G. Peng J. Ran F. Wang Z. et al. (2022). Identifying and monitoring students’ classroom learning behavior based on multisource information. *Mobile Informat. Syst.* 2022:9903342. 10.1155/2022/9903342

[B29] Zhao J. Zhu H. (2023). CBPH-Net: a small object detector for behavior recognition in classroom scenarios. *IEEE Transact. Instrument. Meas.* 72 1–12. 10.1109/TIM.2023.3296124

[B30] Zhao Y. Lv W. Xu S. Wei J. Wang G. Dang Q. et al. (2024). “Detrs beat yolos on real-time object detection,” in *Proceedings of the IEEE/CVF Conference on Computer Vision and Pattern Recognition*, (Vancouver, BC).

[B31] Zhou J. Ran F. Li G. Peng J. Li K. Wang Z. (2022). Classroom learning status assessment based on deep learning. *Math. Probl. Eng.* 2022:7049458. 10.1155/2022/7049458

[B32] Zhu W. Yang Z. (2024). CSB-YOLO: a rapid and efficient real-time algorithm for classroom student behavior detection. *J. Real-Time Image Process.* 21:140. 10.1007/s11554-024-01515-8

[B33] Zou Z. Chen K. Shi Z. Guo Y. Ye J. (2023). Object detection in 20 years: a survey. *Proc. IEEE* 111 257–276. 10.48550/arXiv.1905.05055

